# A Study of Residue Correlation within Protein Sequences and Its Application to Sequence Classification

**DOI:** 10.1155/2007/87356

**Published:** 2007-09-10

**Authors:** Chris Hemmerich, Sun Kim

**Affiliations:** 1 Center For Genomics and Bioinformatics, Indiana University, 1001 E. 3rd Street, Bloomington, Indiana, IN 47405-3700, USA; 2 School of Informatics, Center for Genomics and Bioinformatics, Indiana University, 901 E. 10th Street, Bloomington, Indiana, IN 47408-3912, USA

## Abstract

We investigate methods of estimating residue correlation within protein sequences. We begin by using mutual information (MI) of adjacent residues, and improve our methodology by defining the *mutual information vector* (MIV) to estimate long range correlations between nonadjacent residues. We also consider correlation based on residue hydropathy rather than protein-specific interactions. Finally, in experiments of family classification tests, the modeling power of MIV was shown to be significantly better than the classic MI method, reaching the level where proteins can be classified without alignment information.

## 

[1,2,3,4,5,6,7,8,9,10,11,12,13,14,15]
